# Prevalence and risk factors for hyperthyroidism in Irish cats from the greater Dublin area

**DOI:** 10.1186/s13620-017-0113-x

**Published:** 2018-01-15

**Authors:** Laura Bree, Barbara A. Gallagher, Robert E. Shiel, Carmel T. Mooney

**Affiliations:** 1 Section of Small Animal Clinical Studies, School of Veterinary Medicine, University College Dublin, Belfield, Dublin 4, Ireland; 2 Present Address: Chestergates Veterinary Specialists, Telford Court Chestergates Roads Chester, Cheshire, CH1 6LT UK

**Keywords:** Feline, Hyperthyroidism, Prevalence, Ireland, Risk, Epidemiology, Thyroid

## Abstract

**Background:**

Hyperthyroidism is common in older cats. Prevalence varies geographically, but is anecdotally considered low in Ireland. The aim of this study was to document prevalence of hyperthyroidism in older cats in the greater Dublin area of Ireland and to assess environmental and clinical associations for development and identification of the disease.

**Methods:**

Primary-care veterinary practices were requested to select cats aged 10 years or older where blood sampling was being performed for health screening or clinical investigations. Surplus serum/plasma samples were submitted to University College Dublin Diagnostic Endocrine Laboratory for total thyroxine (T_4_) measurement. Cats were classified as hyperthyroid, equivocal or euthyroid based on a total T_4_ concentration (reference interval, 15–60 nmol/L), of >60 nmol/L, 30–60 nmol/L or <30 nmol/L, respectively. Simultaneous free T_4_ or repeat (after 4–6 weeks) total T_4_ measurement was recommended in all equivocal cases. Animals receiving treatment for hyperthyroidism were excluded. A questionnaire completed by the client and veterinarian detailing historical and physical information was also required. Associations between categorical variables were analysed by Chi-square or Fisher’s exact test and odds ratio (OR) calculated. A *P* value of <0.05 was considered statistically significant.

**Results:**

Samples were submitted from 507 cats including 107 (21.1%) hyperthyroid, 54 (10.6%) equivocal and 346 (68.2%) euthyroid. The presence of goitre (*P* < 0.0001), tachypnoea (*P* = 0.0378), tachycardia (*P* = 0.002), polyphagia (*P* = 0.0003) and weight loss (*P* < 0.0001) were significantly associated with hyperthyroidism. Cats with goitre were more likely to be diagnosed as hyperthyroid [OR 2.85, (95% CI 1.75–4.62] compared to those without. However, goitre was only palpated in 40 of 102 (39.2%) hyperthyroid cats. Increasing age was the only significant (*P* < 0.002) risk factor for development of hyperthyroidism. A relationship between hyperthyroidism and sex, breed, lifestyle, parasite control, vaccination status or feeding habits was not identified.

**Conclusions:**

Hyperthyroidism is not uncommon in Irish cats. Age was the only significant risk factor for its development. The high proportion of hyperthyroid cats without palpable goitre (> 60%) may reflect failure to detect goitre and account for the perceived low prevalence of this condition in Ireland.

**Electronic supplementary material:**

The online version of this article (10.1186/s13620-017-0113-x) contains supplementary material, which is available to authorized users.

## Background

Hyperthyroidism is a disorder of older cats, resulting from excess circulating concentrations of triiodothyronine (T_3_) and, or thyroxine (T_4_) [1, 2] produced by an abnormally functioning thyroid gland. Histopathologically, most affected cats have benign adenomatous hyperplasia (adenoma) with only a small proportion having thyroid carcinoma [3, 4]. The median age at diagnosis is typically approximately 13 years and it is uncommonly diagnosed in cats less than 10 years of age [5].

Not only is hyperthyroidism recognized as the most common endocrinopathy in cats but also as one of the more frequently diagnosed disorders in small animal practice in the UK, USA, Australia, Japan and several other European countries. It was first definitively diagnosed in 1979 and has since been recognized with increasing frequency [3, 6, 7]. In the USA, a 20-fold increase in prevalence was reported from 0.1% in 1978–1980 to 2% in 1993–1997 [8]. Similarly, a 13-fold increase in prevalence has been reported in Germany from 0.2% between 1987 and 1994 to 2.6% in 1998 [9]. Other epidemiological studies have estimated prevalence in these and other countries and suggest a geographical variation. For instance, prevalence in older cats has been reported as 8.9% in 2002 for Japan [10], 11.4% in 2006 [11] and 12.3% in 2016 [12] for Germany, 3.9% in 2008 for Hong Kong [13], 20.1% in 2014 for Poland [14], 9.0% in 2014 for Portugal [15] and 7.0% in 2016 for South Africa [16]. A cumulative yearly incidence rate of 11.9% was reported in London, UK compared to 1.5% in Spain [17]) and in a similar UK study was suggested to be 7.4% [18]. Perhaps most relevant are the recent larger studies estimating prevalence of hyperthyroidism in primary-care veterinary practices in the UK. The prevalence in a population of 3584 cats unclassified by age was 3.0% [19]. In another study of 95,629 cats, the apparent prevalence was 2.4% but 8.7% in cats greater than 10 years of age [20]. Although direct comparisons between these studies is complicated by differing populations, varying age ranges and variable cut-offs for diagnosing hyperthyroidism and classification of equivocal cases, it appears that where hyperthyroidism is uncommon it has a prevalence of less than 4% and where more common, one of approximately 10% or more. Despite its close proximity to the UK and similar cultural and socio-economic status, the prevalence of feline hyperthyroidism in Ireland has anecdotally been considered as low.

Undoubtedly cats are living longer today compared to decades ago and hyperthyroidism is a disease associated with ageing. However, an increase in the aged population alone does not explain the significant rise in prevalence over the last 30+ years as the prevalence of hyperthyroidism has increased at a rate exceeding that of diabetes mellitus and chronic kidney disease, both of which also occur in older cats [8]. Increased awareness and improved diagnostic capabilities have contributed to the increase in prevalence but again are unlikely to be solely responsible for the dramatic increase observed in the recent past.

Although the clinical and pathological features of this disease have been well-described, the exact cause(s) remains elusive [2, 21, 22]. Many hypotheses have been explored such as immunological, infectious, nutritional, environmental and genetic factors, but a single dominant factor, other than advanced age, has not yet been identified. The most widely studied risk factors fall into the two broad categories of nutritional and environmental. The first large epidemiological study published in 1988 found an association between the development of hyperthyroidism and feeding canned food in the 5 years preceding diagnosis [23]. Such an association has been highlighted by almost all other studies where it has been addressed [3, 8, 12, 14, 16, 23–26] and in some instances, has been refined by certain flavours (e.g. fish, liver or giblet) [23, 25] and food specifically from ring pull (pop top) or aluminium cans [8, 12]. Other associations, variably reported in different studies have included exposure to fertilizers, herbicides, pesticides or flea products [3, 26], living indoors [3, 14], using cat litter [23, 24] and being female [8, 12, 26]. Undoubtedly, there are many potential thyroid disruptors that cats may be exposed to, either through their diet or from the environment. Several factors have since been implicated in the development of hyperthyroidism including dietary iodine intake [27], soy isoflavone excess [28, 29], exposure to bisphenol A from pop top or ring pull cans [8, 30] and polybrominated diphenyl ethers (PBDEs) from the environment [31–33]. Overall the association between diet and development of hyperthyroidism is controversial and exemplified by the presence of the same potential dietary risk factors in areas of low prevalence of the disease [13].

Numerous studies have also suggested that being purebred and particularly Siamese, Himalayan or Burmese is associated with lower odds of developing the disease [3, 19, 23, 24]. This suggests that whatever the cause of the disease, there is at least an underlying genetic predisposition for its development.

The aims of this study were to determine the prevalence of hyperthyroidism in Irish cats in the greater Dublin area and to determine potential risk factors associated with the disease in this geographical location.

## Methods

### Sample size

A pilot questionnaire for veterinarians was first conducted at a national veterinary conference (University College Dublin (UCD) Veterinary Hospital Conference 2010) to more accurately estimate the perceived prevalence of hyperthyroidism in Ireland. Results of that pilot study confirmed the perceived low prevalence of the disorder. In order to calculate an appropriate sample size, in a population where a low prevalence was suspected, an estimated prevalence was calculated using the results of a similar study in Hong Kong [13], where a prevalence of 3.9% was identified using a test (total T_4_ estimation) with 98.5% sensitivity. This prevalence was utilized to calculate an appropriate sample size using the formula:

$$ Sample size=\frac{Z^2P\ \left(P-1\right)}{d^2} $$

Where Z = confidence interval, *P* = estimated prevalence, d = precision. A precision of <5% of the estimated prevalence was advised where estimated prevalence was <5% [34]. A descriptive cross-sectional study was designed based on the results of the pilot study. Sample size was calculated to be approximately 500 cats, for a prevalence of 3.9% and 95% confidence interval (CI), with a predicted precision of 2.6%.

### Case selection

A list of 45 first opinion veterinary practices in the greater Dublin area was compiled using the Veterinary Council of Ireland’s Statutory Premises Accreditation Scheme (www.vci.ie) and veterinarians were invited to participate in the study between June 2011 and July 2012. Each practice received by mail a description of the study, questionnaires for completion by the owner and veterinarian and sample submission requests (Additional file 1). Two one-page questionnaires were designed for the owner and veterinarian, specifically based on previously reported risk factors (e.g. lifestyle, use of a litter box, exposure to ring pull cans). The information collected from the owner included age, breed, sex, length of time in possession, number of cats in the household, vaccination status, endo- and ectoparasiticide treatments and preparations, environmental surroundings, a detailed history of feeding habits including dry/wet proportion fed, type of container used (e.g. ring pull, regular or pouch) and preferred brand or flavouring. Information collected from the veterinarian included sample type, reason for veterinary attendance, and clinical features. Veterinarians were asked to describe the cat as ‘sick’ or ‘healthy’. Cats were eligible for inclusion in the study if they were ≥ 10 years of age, both owner and veterinarian questionnaires were completed and blood sampling was being performed as part of their routine investigations. Exclusion criteria included cats already diagnosed as hyperthyroid or where they were receiving anti-thyroid medication. The study was approved by the UCD Academic Research and Ethics Committee (AREC-P-11-20-Mooney).

### Sample collection and analysis

Blood samples were collected by jugular venipuncture, transferred into plain or lithium heparinized tubes and centrifuged prior to separating the serum or plasma. All samples were cooled to 4°C, transported to UCD Veterinary Diagnostic Endocrine Laboratory and stored at 4°C for a maximum of 4 days prior to total T_4_ measurement on site using an immunoassay previously validated for use in cats (Immulite 1000 canine total T_4_, Siemens) [35]. Where volume allowed, aliquots were stored at −20°C for future free T4 analysis, if required. The established laboratory reference interval for total T4 was 15–60 nmol/L. Cats were classified as hyperthyroid, equivocal or euthyroid based on a total T_4_ concentration of >60 nmol/L, 30–60 nmol/L or <30 nmol/L, respectively. For the purposes of the study, all cats with a total T4 concentration below the reference interval (<15 nmol/L) were included in the euthyroid group. For this assay, the limits of detection were 6.4 and 193.0 nmol/L and all values below or above were assigned as those values for the purposes of statistical analyses. Repeat measurement of total T_4_ after 4–6 weeks, or simultaneous free T_4_ measurement (reference interval 10–50 pmol/L) (Free T_4_ by equilibrium dialysis, Antech Diagnostics performed at Nationwide Diagnostic Laboratories, UK) was recommended in all equivocal cases.

### Statistical analysis

The prevalence of hyperthyroidism, with exact binomial 95% CI, was calculated for all cats combined, for cats classified as healthy and for cats classified as sick, using the Wilson interval test [36]. Data were examined for normality using the Shapiro Wilk test. Non-parametric data were reported as median (range) and parametric data were reported as mean ± standard deviation (SD). Breed was analysed by classification as crossbreed or purebred. To determine whether age should be treated as a continuous or categorical variable, it was examined for linearity (log odds of hyperthyroid cats) and as no linear trend was established, it was treated categorically. Age was therefore modeled with three categories (≥ 10 - < 12 years, ≥ 12 - < 14 years and ≥ 14 years). Equivocal cases were excluded from all association analysis. Univariate association analysis was performed using a two-tailed Fisher’s exact test. Multivariate association analysis was performed using a Chi-square test for those variables with more than one category (e.g. indoor/outdoor/access to outdoor). Thereafter, any significant predictors resulting from these analyses were entered into a binary logistic regression model to further investigate their effects on the presence of hyperthyroidism.

The Mann Whitney U test was used to compare the initial total T_4_ concentration between healthy and sick hyperthyroid cats and between healthy and sick euthyroid cats. This test was also used to compare total T_4_ concentrations in hyperthyroid cats with or without palpable goitre. In all cases a *P* value <0.05 was considered significant.

## Results

The study population consisted of 507 cats, from 35 first opinion practices in the greater Dublin area. Each practice submitted a median of 5 (1–86) samples. There were 196 males, 271 females and 40 where gender was unspecified. Breeds included domestic shorthair (DSH) (*n* = 434), domestic longhair (DLH) (*n* = 27), British Blue (*n* = 4), Persian (*n* = 3), Siamese (*n* = 3), Burmese (*n* = 2), Birman (*n* = 1) and 33 where breed was not specified. The median age of the cats was 13 (range 10–21) years.

Of the 507 cats, 395 presented for various illnesses, 70 for annual health check, 34 for routine vaccination and reasons were not recorded for 8 cats. Thus, the study comprised 395 (77.9%) cats classified as ‘sick’ and 104 (20.5%) classified as ‘healthy’. Hyperthyroidism was definitively diagnosed after a single T_4_ measurement in 100 cats. Seven further cats were subsequently identified as hyperthyroid by repeat total T_4_ (*n* = 3) or free T_4_ (*n* = 4) measurement giving a total of 107 hyperthyroid cats and a prevalence for this disease of 21.1% (95% CI, 17.7–24.8). Of the remainder, 318 cats were considered euthyroid after a single T_4_ measurement. A further 28 cats were confirmed as euthyroid by repeat total T_4_ measurement (*n* = 2) or free T_4_ analysis (*n* = 26) resulting in 346 (68.2%) euthyroid cats. In total 89 cats initially had an equivocal total T4 concentration but 7 (7.9%) and 28 (31.5%) were subsequently diagnosed as hyperthyroid or euthyroid, respectively. Thus, 54 (10.7%) cats in total were considered equivocal including five cases where total T_4_ was repeat tested but remained within the equivocal range.

**Fig. 1:**
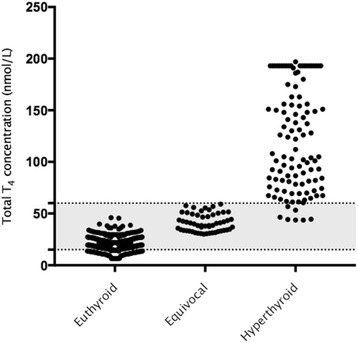
Initial total thyroxine (T4) concentrations in cats classified as euthyroid, equivocal and hyperthyroid. The reference interval for total T4 (15–60 nmol/L) is indicated by the shaded area. Values above 193.0 and below 6.4 nmol/L are all assigned as those values, respectively

Total T_4_ concentrations in the different groups of cat are presented in Fig. 1. The median initial total T_4_ concentration for hyperthyroid cats was 108.0 (43.7–193.0 nmol/L including the 7 (6.5%) equivocal cases on initial measurement. Within the hyperthyroid group 80 (74.8%) cats were classified as sick, 23 (21.5%) as healthy and 4 (3.7%) were not classified. Median initial total T_4_ concentration for healthy hyperthyroid cats (101.0 (43.7–193.0) nmol/L) was not significantly different (*P* = 0.153) from sick hyperthyroid cats (125.0 (44.5–193.0) nmol/L). Median initial total T_4_ concentration for euthyroid cats was 20.9 (6.4–46.0) nmol/L. For this group, 273 (78.9%) were classified as sick, 70 (20.2%) as healthy and 3 (0.9%) were not classified. Sixty-one (17.6%) of this group had initial total T_4_ concentration below the reference interval (< 15.0 nmol/L). Of these cats, 49 (80.3%) were classified as sick, 11 (18.0%) as healthy and one (1.7%) was not classified. There was a significant association (*P* < 0.0001) between a total T_4_ concentration < 15.0 nmol/L and being sick for all cats. There was no significant difference between the prevalence of hyperthyroidism in cats classified as sick (20.3%; 95% CI, 16.5–24.4) and healthy (22.1%; 95% CI, 15.2–31.0).

There was a significant positive association between hyperthyroidism and increasing age (*P* < 0.002) but no association with sex, breed, lifestyle, parasite control, vaccination status or feeding habits (access to dry food or ring-pull canned food) (Table 1). When age was entered into the logistic regression model, cats between 10 and 12 years old were at reduced odds of hyperthyroidism compared to cats greater than 14 years of age (OR 0.47, 95% CI 0.26–0.84, *P* = 0.0094), while cats greater than 14 years of age were at significantly increased odds of hyperthyroidism compared to cats less than 12 years of age (OR 2.18, 95% 1.39–3.44, *P* = 0.0007). Of the euthyroid and hyperthyroid cats with breed information reported (*n* = 423), the majority (*n* = 411 (97.2%)) was DSH or DLH. Too few other breeds were included to allow meaningful statistical analyses but there was no significant association between hyperthyroidism and being crossbreed or purebred. The presence of goitre, increased respiration rate, tachycardia, polyphagia and weight loss were all significantly associated with a diagnosis of hyperthyroidism (Table 2). When heart rate was further examined using logistic regression, cats with heart rate > 240 bpm were at significantly increased odds of hyperthyroidism compared to cats with a heart rate < 200 bpm (OR 3.59, 95% CI 1.74–7.44,*P* = <0.0001). Diarrhoea, vomiting and presence of cardiac murmur were not significantly associated with disease.

**Table 1:** Association of signalment and historical data with hyperthyroidism in 507 cats 10 years and older presented to primary care veterinary practices in the greater Dublin area

		All cats (*n* = 507)		Euthyroid cats (*n* = 346)		Hyperthyroid cats (*n* = 107)				
Variable		n^a^	%	n^a^	%	n^a^	%	OR	CI	P
Age	10 - < 12	121	24.6	94	28.1	16	15.4	–	–	–
	≥12 - <14	130	26.5	96	28.7	23	22.1	–	–	–
	≥14	240	48.9	145	43.3	65	62.5	–	–	0.0019^b^
Sex	Male	196	42.0	141	44.3	39	38.6	0.78	0.49–1.24	0.3563
	Female	271	58.0	177	55.7	62	61.4			
Breed	Crossbreed	461	97.3	316	96.9	95	97.9	1.50	0.32–6.97	0.747
	Purebreed	13	2.7	10	3.1	2	2.1			
Lifestyle	Indoor only	105	21.2	74	21.9	16	15.1	–	–	–
	Outdoor only	23	4.6	16	4.7	5	4.8	–	–	–
	Indoor and Outdoor	368	74.2	248	73.4	83	79.8	–	–	0.349
Parasite control	Frequent or infrequent	350	76.62	244	77.7	68	72.3	0.75	0.44–1.26	0.3317
	Never	107	23.4	70	22.3	26	27.7			
Vaccination	Frequent or infrequent	346	76.5	241	77.7	67	72.0	0.73	0.43–1.24	0.2669
	Never	106	23.5	69	22.3	26	28.0			
Feeding habits										
Access to dry food	Yes	310	63.9	208	63.0	69	67.6	1.22	0.76–1.96	0.411
	No	175	36.1	122	37.0	33	32.4			
Fed ring pull	Yes	179	52.0	119	50.6	39	52.7	1.08	0.64–1.83	0.7907
	No	165	48.0	116	29.4	35	47.3			

^a^refers to number of cases where recorded

^b^logistic regression analysis (see text for exact results)

Chi-squared was used to analyse data with more than two categories (age and lifestyle). Fisher’s exact test with odds ratio calculation was used for all other analyses

**Table 2:** Association of clinical signs with hyperthyroidism (T_4_ > 60 nmol/L) in 507 cats 10 years and older presented to primary care veterinary practices in the greater Dublin area

		All cats (*n* = 507)		Euthyroid cats (*n* = 346)		Hyperthyroid cats (*n* = 107)				
Variable		n^a^	%	n^a^	%	n^a^	%	OR	95% CI	P
Goitre (L, R or Both)	Yes	112	23.1	61	18.5	40	39.0	2.85	1.75–4.62	<0.0001
	No	373	76.9	269	81.5	62	60.8			
Vomiting	Yes	148	69.5	99	29.9	40	39.6	1.53	0.97–2.44	0.0696
	No	337	30.5	232	70.1	61	60.4			
Diarrhoea	Yes	54	11.3	31	9.0	15	15.0	1.68	0.86–3.25	0.1403
	No	424	88.7	295	85.3	85	85.0			
Respiration rate	Normal	377	74.4	267	77.2	70	70.7	1.72	1.04–2.88	0.0378
	Increased	106	20.9	64	18.5	29	27.1			
Murmur	Yes	124	26.1	79	24.2	31	32.3	1.49	0.91–2.45	0.1451
	No	351	73.9	247	75.8	65	67.7			
Heart Rate	< 200	280	58.6	201	61.7	50	50.5	–	–	–
	200–240	158	33.1	106	32.5	32	32.3	–	–	–
	> 240	40	8.4	19	5.8	17	17.2	–	–	0.001^b^
Polyphagia	Yes	162	34.7	98	30.4	48	51.1	2.38	1.5–3.81	0.0003
	No	305	65.3	224	69.6	46	48.9			
Weight loss	Yes	328	68.0	208	63.2	87	85.3	3.37	1.86–6.09	<0.0001
	No	154	32.0	121	36.8	15	14.7			

^a^refers to number of cases where recorded

^b^logistic regression analysis (see text for exact results)

Chi-squared was used to analyse data with more than two categories (respiration rate and heart rate). Fisher’s exact test with odds ratio calculation was used for all other analyses

**Fig. 2:**
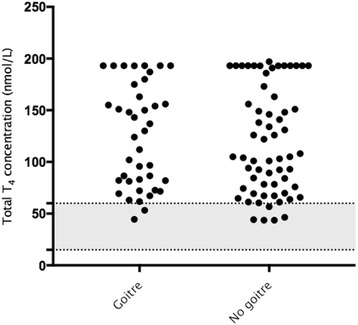
Serum total thyroxine (T_4_) concentration in 40 and 60 hyperthyroid cats with or without goitre. The reference interval for total T_4_ (15–60 nmol/L) is indicated by the shaded area. Values above 193.0 and below 6.4 nmol/L are all assigned as those values, respectively

A total of 112 (23.1%) of 485 cats were reported as having goitre. Overall goitre was recorded in 40 of 102 (39.2%) hyperthyroid cats, 11 of 53 (20.8%) equivocal and 61 of 330 (18.4%) euthyroid cats. There was no significant difference (*P* = 0.645) between the initial total T_4_ concentration in hyperthyroid cats with palpable goitre (127.0 (range 44.5–193.0) nmol/L) compared to those with no palpable goitre (105.0 (range 43.7–193.0) nmol/L) (Fig. 2). Of the 40 hyperthyroid cats with goitre, 21 (52.2%) were unilateral, whilst 19 (47.5%) were bilateral. There was no significant difference (*P* = 0.469) between the detection of goitre and health status of the hyperthyroid cats.

## Discussion

The prevalence of hyperthyroidism in cats older than 10 years of age presenting to primary-care veterinary practices was greater than 20% in the present study. This prevalence exceeds that of 3.9% in Hong Kong where the disease is considered uncommon [13]. It equates more to prevalences between 7 and 20.1% in older cats reported in Japan, Germany, Portugal, Poland, England and South Africa (10, 11, 12, 15, 14, 19, 20, 16], where the disease is considered relatively common. Although the prevalence may appear even higher than in some of these studies, direct comparisons are difficult because of differences in populations tested, inclusion and exclusion criteria used and thyroid hormone concentration cut-offs employed for diagnosing hyperthyroidism. It could be argued that the study design introduced inherent bias reflected by this high prevalence. Practitioners may have been more willing to enter into the study to obtain a diagnosis of hyperthyroidism free of charge in cats more likely to have the disease. Certainly, one practice only submitted a single sample from a cat later proven to be hyperthyroid. However, most practices submitted several samples from a range of both sick and healthy cats and those diagnosed as euthyroid and hyperthyroid. Overall the results of the study confirm that hyperthyroidism, contrary to anecdotal supposition, is frequently encountered in cats within the greater Dublin area of Ireland.

Achieving an accurate diagnosis of hyperthyroidism was essential for this study. In the laboratory used, a cut-off of 60.0 nmol/L was employed to confirm a diagnosis. Although individual laboratory reference intervals vary, measurement of total T_4_ is considered to be highly specific for hyperthyroidism especially when, as in this study, higher cut-off values are used [37, 38]. On the other hand, total T_4_ concentrations may be within reference interval in between 5 and 10% of all hyperthyroid cats and in up to 40% of those considered mildly affected [37, 38]. In general, such values are within the mid to high end of the reference interval and may reflect the co-existence of hyperthyroidism and non-thyroidal illness (NTI) [39] or, more commonly, early or mild thyroid disease [37, 38]. Therefore, in the present study, hyperthyroidism could not be confidently eliminated with a total T_4_ concentration between 30 and 60 nmol/L. Initially approximately 18% of cats were classified as equivocal, comparable to 14% of 197 cats [15] and 15.6% of 302 cats [16] where an equivocal category was specifically reported. A recommendation for simultaneous free T_4_ analysis or repeat total T_4_ estimation was made to further elucidate thyroid status in these cats as has been recommended elsewhere [12, 15, 16]. However, the former was often not possible because of small sample volume or concerns over inappropriate sample handling that may have affected validity of free T_4_ analysis in the samples submitted. The possibility of prolonged storage during submission was a particular concern as it is known that free T_4_ concentrations increase by approximately 50% after 5 days at 37 °C, at least when assessed by equilibrium dialysis [38]. On the other hand, repeat testing was not always possible because of cats lost to further follow-up. How likely equivocal cases are to be ultimately classified as hyperthyroid is largely unknown but could significantly increase the prevalence of the disease if considered highly likely. In the present study, additional (free T_4_) or repeat (total T_4_) testing was only performed in approximately 45% of these equivocal cases and resulted most commonly (70.0%) in reclassification as euthyroid and less commonly as hyperthyroid (17.5%) or persistently equivocal (12.5%). In other studies, highlighting equivocal cases, only 1 of 47 (2.1%) overall [16] and 5 of 46 (10.9%) [12] cats with supportive clinical signs were reclassified as hyperthyroid. Measurement of canine thyroid stimulating hormone (cTSH) could have been used to support a diagnosis of hyperthyroidism in equivocal cases. However, it is neither wholly sensitive or specific for diagnosing hyperthyroidism [40] and was not routinely used at the time of the study. It has been used elsewhere when evaluating equivocal cases but was undetectable in 21 cases where free T_4_ concentration was only elevated in one [16], suggesting it should be interpreted cautiously in this group of cats. A possible advantage of measuring cTSH is that it provides some information on the likelihood of developing hyperthyroidism in the near future [18]. However, the current study was evaluating point prevalence and therefore future development of hyperthyroidism was not particularly relevant. Overall because there were so few equivocal cases and that most of those retested proved euthyroid, this group is unlikely to significantly impact the overall prevalence rate.

Many euthyroid cats had a total T4 concentration below the reference interval. Further investigations to eliminate hypothyroidism were not performed, but such a diagnosis was considered unlikely and not truly relevant to the aims of the study. Naturally occurring adult-onset hypothyroidism is extremely rare with only four case reports in the literature [41–44]. On the other hand, non-thyroidal illness is a well-known cause of suppressed total T4 concentrations in cats [5]. The majority (approximately 80%) of cats with low total T4 concentrations were classified as sick in the current study, supporting a diagnosis of non-thyroidal illness as a potential cause. However, approximately 20% of these cats were reportedly healthy. It is possible that, given the limitations of a routine physical examination, these cats were incorrectly identified as healthy or that for some, the low values simply reflect normal variation.

Hyperthyroidism is a disease of aged cats and it is therefore not surprising that cats older than 14 years were over twice as likely to have hyperthyroidism compared to the younger cats. This is consistent with all other studies where age has been evaluated [8, 12–14, 16, 18, 25]. However, whilst recognizing the importance of advancing age, it is also recognized that ageing alone is not responsible for the increasing prevalence of hyperthyroidism.

Despite targeting similar risk factors for hyperthyroidism, the results of the current study did not always support other published reports. There were more female (61.4%) than male (38.6%) hyperthyroid cats in the current study, but there was no statistically significant difference in prevalence of hyperthyroidism between them. Most other previous studies where gender was examined also report no sex predilection [3, 13, 14, 16, 19, 24]. This is in contrast to three separate studies reporting a higher prevalence of hyperthyroidism in female cats [8, 12, 26] and one suggesting an increased association in male cats [11] In humans, toxic multinodular goitre, with which the feline condition is likened to, is many more times common in females, although the reasons remain unclear [45]. The variable and different sex predispositions in the reports of feline hyperthyroidism would question the true significance of this as a risk factor for the disorder in this species.

Being purebred and particularly Siamese, Himalayan, Burmese or Persian has been associated with a decreased risk of hyperthyroidism suggesting a genetic predisposition to the disease [3, 19, 23, 24, 26]. In a study from Hong Kong, being non-DSH was significantly associated with being hyperthyroid but this might reflect a large percentage of DSH cats in that particular region being of oriental descent and could actually support the previously reported breed predispositions [13]. By contrast, in the current study, there was no significant association between hyperthyroidism and being purebred or crossbred. There were only eight cats of oriental origin, of which one was hyperthyroid. The small number of purebred cats (< 3%) prevented any meaningful analysis of a specific breed predilection but also may have influenced the lack of association found for breed overall. How the proportion of purebred cats presented during this study reflects the distribution of cats in the wider Irish cat population is largely unknown as reliable figures are not available.

The association between different environmental factors and feline hyperthyroidism is variable in most studies and made no more well defined based on the results of the current study. Of the cats evaluated herein, the majority was indoor with access to outdoors, a minority was exclusively indoor with only a few being completely outdoors. There was no significant association between hyperthyroidism and lifestyle as assessed by living indoors and outdoors. This contrasts with a reported significant association between hyperthyroidism and living strictly indoors noted in two separate studies [3, 14] or presumably having indoor access as defined by use of cat litter [23, 24] or sleeping on the floor [26]. However, many other studies found no association between a mostly indoor lifestyle and hyperthyroidism [12, 13, 16, 25]. Undoubtedly, living strictly indoors could increase exposure to endocrine disruptors such as PBDEs that have been used as flame retardants. Their involvement in the aetiology of hyperthyroidism has recently been disputed, at least in Australian cats [46]. However, several other studies have suggested that PBDEs may be involved in the aetiopathogenesis of the disease, at least in Europe and California [31–33]. Alternatively, clients owning cats that are kept predominantly indoors may be more observant of clinical signs associated with hyperthyroidism and thus more likely to present for veterinary attention. This may be supported by the reported association between being insured and hyperthyroidism [19] as insured cats are more likely to attend for veterinary attention more regularly and to undergo more diagnostic investigation.

Regular exposure to topical ectoparasiticides has also been associated with an increased risk of developing hyperthyroidism in some previous studies [3, 26]. However, in line with the current results, this association has not been identified in multiple other studies [12, 13, 16, 25]. Thus, the role of exposure to parasiticides and the development of feline hyperthyroidism is probably minimal. This presumably also applies to other environmental chemicals such as fertilizers and herbicides that have been associated with hyperthyroidism in only one previous study [3].

Multiple studies have identified an increased risk of hyperthyroidism associated with an increased proportion of canned or wet food in the diet [3, 8, 12, 14, 16, 23–26], particular flavours of canned food [23, 25] and types of packaging (easy open, ring pull or pop top tins) [8, 12]. Only one previous study failed to identify an association with feeding canned food [26] similar to the results of the current study. However, evaluating any association between types of food and hyperthyroidism was particularly difficult in the current study. There was a mixed response to this section of the questionnaire as the majority of respondents fed a combination of pouches, cans and dry food, and/or failed to specify the type of packaging. Thus, it was only possible to examine two groups considered large enough; cats that had access to ring pull cans versus those that did not, and cats that had dry food as part of their diet versus those fed exclusively wet food. There was no significant association in either case but this only included a proportion of the cats investigated. Thus, the role of diet in the development of hyperthyroidism cannot be dismissed from these results, be it, as suggested elsewhere, related to iodine content [27], soy isoflavone excess [28, 29], exposure to bisphenol A from pop top or ring pull cans [8, 30] or other as of yet unidentified factors. The variable risk factors identified in this and other studies suggests that a much larger multicenter study should be performed to truly identify those that are significant as recommended elsewhere [16, 47].

Within the current study, the clinical findings in hyperthyroid cats were not unexpected and have been reported extensively elsewhere [1–3, 18, 22]. Within this group, weight loss, tachypnoea, and polyphagia were noted in the majority of cases whilst goitre, cardiac murmur, increased heart rate and diarrhoea occurred in less than 50% of cases. There was a significant association between weight loss, tachypnoea, polyphagia, tachycardia and goitre and hyperthyroidism similar to previous reports [16] and suggesting that the presence of these signs should specifically prompt investigation for hyperthyroidism. Despite vomiting and diarrhoea described as relatively common clinical signs of hyperthyroidism [2], there was no association between these and thyroid dysfunction in the current study as reported elsewhere [26]. This presumably relates to the frequency (approximately 32%) with which vomiting was recorded in the cats overall and the fact that there are many wide-ranging differentials for such a presenting problem. On the other hand, a lack of association with diarrhoea may have reflected the low numbers of cases (just over 10%) with this complaint overall. Similarly, there was no association with the presence of a cardiac murmur. However, such was only identified in approximately one third of the hyperthyroid cats where it has been recognized in over half of affected cats in other studies [48]. This may reflect difficulties in auscultating cardiac murmurs during primary-care veterinary practice consultations and warrants further evaluation.

Most notably in the present study, although there was an association between detection of goitre and hyperthyroidism, less than half of the hyperthyroid cats were reported as having the same. Additionally, just less than half of these were reported to have bilateral disease, there was no significant difference in total T_4_ concentration between hyperthyroid cats with or without goitre and no significant difference between the detection of goitre and the health status of these cats. There are numerous studies reporting that bilateral disease is more common in hyperthyroid cats and that increasing severity of hyperthyroidism correlates with the degree of thyroid gland enlargement. It has been reported elsewhere that in primary-care veterinary practice, goitre was accurately detected in over 90% of hyperthyroid cats, with the majority (87%) having bilateral disease [49]. In another study, all hyperthyroid cats had detectable goitre again with the majority being bilateral [50]. Both of these studies demonstrated increasing size of affected thyroid lobes with increasing total T_4_ concentration. Of particular note is that bilateral goitre was evident in over 60% of 2096 hyperthyroid cats as demonstrated by thyroid scintigraphy [51] confirming its preponderance in this disease. It appears most likely that the veterinarians contributing to the current study failed to detect goitre in hyperthyroid cats. This was not due to uncertainty as in only 14% of the hyperthyroid cats was the presence of goitre listed as ‘unsure’. Undoubtedly, the low detection rates found in this study may account for the perceived low prevalence of feline hyperthyroidism in Ireland. Indeed, in South Africa where the prevalence of hyperthyroidism was also perceived as low before being specifically investigated, goitre was only detected in 2 of 12 hyperthyroid cats [16].

Goitre was also detected in between 20 and 25% of euthyroid and equivocal cases in the current study. Palpation of small cervical nodules, presuming a goitre, is not uncommon and has been previously reported in euthyroid cats [49, 50]. Such cervical nodules may represent other pathologies such as parathyroid enlargement. If truly representing goitre, it would be interesting to follow such cats to determine if hyperthyroidism develops with time. Certainly, in one study, half of the euthyroid cats with palpable goitre went on to develop increased total T4 concentrations, but only six cats were evaluated [49]. It would appear prudent to monitor these cases regularly over time.

## Conclusion

Hyperthyroidism is not uncommon in Irish cats and similar to other studies, advancing age is a significant risk factor. However, no associations between previously reported environmental and nutritional risk factors and feline hyperthyroidism were detected in the present study. The high proportion of hyperthyroid cats with no palpable goitre (>60%) may reflect failure to detect goitre and account for the perceived low prevalence of this condition in Ireland. Large, prospective multi-centre studies are required to more fully elucidate potential risk factors for hyperthyroidism in cats.

## Additional file

Additional file 1: Study Questionnaire Part 1 (veterinarian) & Part 2 (owner). (PDF 182 kb)
