# Supplementary material for: Prevalence and risk factors for hyperthyroidism in Irish cats from the greater Dublin area
Source: Ir Vet J. 2018 Jan 15;71:2. doi: 10.1186/s13620-017-0113-x (PMC5769238; doi:10.1186/s13620-017-0113-x)
Supplement: Additional file 1: — Study Questionnaire Part 1 (veterinarian) & Part 2 (owner). (PDF 182 kb) [file 13620_2017_113_MOESM1_ESM.pdf]

QUESTIONNAIRE – PART I (TO BE COMPLETED BY VETERINARIAN)

- **1-2ml of blood in lithium heprin** ☐ *(✓ tick here)* or in **serum gel** ☐ *(✓ tick here)* has been submitted.
- Choose a method for results to be relayed to you *(enter the relevant contact information in the space provided)*:

  Telephone ☐ *(✓ tick here)* ____________________________, **fax** ☐ *(✓ tick here)* ____________________________

  or **email** ☐ *(✓ tick here)* ____

Name of **vet**: ____ Date: ________________________

Name of **clinic**:____

I confirm that the cat sampled is **<u>not</u>** under treatment for hyperthyroidism at present ☐ *(✓ tick here)*

Owner name: ____ **Cat** name: ____________________

Reason for visit

- Routine health check ☐
- Vaccination ☐
- Illness ☐ *(specify)* ____

Clinical examination findings

- Has the cat lost weight in the last 3 months? Yes ☐ No ☐
- Is the cat eating excessively? Yes ☐ No ☐
- Heart rate:
  - <200 ☐
  - 200-240 ☐
  - >240 ☐
- Is a cardiac murmur audible? Yes ☐ No ☐
- Respiratory rate:
  - Normal ☐
  - Increased ☐
  - Dyspnoeic ☐
- Does the cat vomit? Yes ☐ No ☐
- Does the cat have diarrhoea? Yes ☐ No ☐
- Are the thyroid glands palpable?
  - Unsure ☐ No ☐
  - Right only ☐ Left only ☐ Both ☐

QUESTIONNAIRE – PART II (TO BE COMPLETED **BY OWNER** AT TIME OF SAMPLING)

Owner name: ______________________________

Cat name: ______________________ **Breed:** ______________________

Sex: Male ☐ Female ☐ Neutered ☐

Age of cat / year of birth: ______________

- *How long have you **owned** your cat?*
  Less than 5 years ☐
  5-10yrs ☐
  More than 10 years ☐
- ***Number** of cats in household:*
  1 ☐
  2 ☐
  More than 2 ☐

Vaccination/worming history

- *Is your cat vaccinated?* Yes ☐ No ☐ Not sure ☐ **Frequency:** Yearly ☐ Infrequently ☐
- *Do you de-worm/de-flea your cat?* Yes ☐ No ☐ Not sure ☐ **Frequency:** Yearly ☐ Infrequently ☐
- *Type of preparation?* Spot-on ☐ Powder ☐ Collar ☐ Spray ☐ Oral ☐

Brand (*if known*) ______________________________

Environment

- *Does your cat live*: always indoors ☐ always outdoors ☐ indoors and outdoors ☐

Diet / food

- *Indicate below which food(s) your cat is fed and also, if mixed, the most commonly used mixture:*

		Most common mix			*Most common mix*
Commercial canned food	☐	☐	Commercial dry food	☐	☐
Commercial pouches	☐	☐	Non-commercial food	☐	☐
Mixture of commercial and noncommercial rations	☐	☐	Mixture of wet and dry food	☐	☐

- *If your cat is fed canned food is it:* Ring-pull can ☐ Tin requiring an opener ☐
- *Does your cat have a favourite brand/flavour that you use most frequently?*

  ____

- *Has your cat had a major change in diet over the last 5 years?* Yes ☐ No ☐ Unsure ☐
- *If you answered '**Yes**' to the above, describe the diet prior to this change*:

  ____

  ____
